# Gemological Characteristic Difference between Colorless CVD Synthetic Diamonds and Natural Diamonds

**DOI:** 10.3390/ma14206225

**Published:** 2021-10-19

**Authors:** Qi Lu, Huaiyu Gong, Qingfeng Guo, Xuren Huang, Jiayi Cai

**Affiliations:** 1 School of Gemology, China University of Geosciences, Beijing 100083, China; qfguo@cugb.edu.cn (Q.G.); h1849599324@163.com (X.H.); q1404980536@163.com (J.C.); 2 Zhengshi Technology Co., Ltd., Shanghai 201700, China; ghy314301@163.com

**Keywords:** CVD synthetic diamond, spectra properties, impurity, fluorescence characteristics

## Abstract

CVD synthetic diamond plays an important role in the jewelry market due to its excellent performance and low cost. In this paper, colorless CVD synthetic diamonds produced by a Chinese company were investigated in detail with their gemological, spectroscopic, and luminescent properties compared with natural colorless diamonds. Compared with natural diamonds, CVD synthetic diamonds have high-order interference color and more apparent abnormal birefringence. The results of infrared spectra indicate that all the CVD samples are classified as type IIa, while the natural samples belong to type Ia. The CVD samples show lamellar growth and mottled luminescence pattern and have blue, orange red, purple red, and blue fluorescence, respectively, while most of the natural samples show blue fluorescence. CVD diamonds show lamellar growth structure, and natural diamonds show irregular ring-like growth structure. Thus, multiple methods combined with analysis are required to distinguish synthetic diamonds from natural diamonds. This work provides an experimental basis for the identification of CVD synthetic diamonds.

## 1. Introduction

CVD (chemical vapor deposition) synthetic diamonds were mainly polycrystalline diamonds in the early years. The single crystal CVD synthetic diamonds were not produced until the end of the 1990s. In the early twenty-first century, Elements Six, Carnegie, Sumitomo, Apollo, and other companies successfully produced single crystal CVD synthetic diamonds [1,2,3]. At the beginning of 2012, the colorless and high-quality synthetic diamonds were successfully produced with microwave plasma chemical vapor deposition by IIa company, Singapore. With the advanced synthetic technology, some Chinese companies have been ranked at the forefront of the synthetic diamond field in the world. How to identify synthetic diamonds from natural diamonds has become an important research subject in gemstone identification.

CVD synthetic diamonds with low color grade always show brown color and often have nitrogen elements in their as-grown state due to their defective structure. Such brown diamonds are generally treated by HPHT (high pressure and high temperature) to render them from near-colorless to colorless. Previous researchers summarized the defects in CVD synthetic diamonds, such as absorption with 3123 cm^−1^ (N-V-H) that may appear in the infrared spectra, 270 nm (isolated nitrogen atom) in the UV visible spectra, 503 nm (H_3_ defect), 575 nm (NV^0^), 637 nm (NV^−^), and 737 nm (Si-V), are often used as the identification characteristics of CVD synthetic diamonds [4,5,6].

One of the most effective analytical methods is optical spectroscopy because it can record the optical characteristics of a gemstone in a way that is quantifiable and reproducible. Synthetic diamond has impurities or extended defects, which is not commonly found in natural diamonds. Certain optical properties can reveal clues about the growth conditions when “the crystal was forming”, and it is significantly different from natural diamonds. Compared with natural diamonds, one of the most typical characteristics of CVD synthetic diamonds is the 737 nm absorption line, which is caused by silicon impurity [5]. The results of photoluminescence analysis showed that the characteristic peaks (736.5 nm and 736.9 nm) present silicon defect (Si-V) in most CVD synthetic diamonds with or without nitrogen impurity. However, an optical center with doublet ZPL at about 737 nm related to Si-V defect is also presented in the spectra of some natural diamonds [7,8,9,10]. Thus, 737 nm double absorption peaks are not a typical identification of CVD synthetic diamonds.

CVD synthetic diamonds produced by some companies such as Gemsis Company and Elements Six Company [11,12] had silicon-vacancy center (Si-V), indicating the presence of silicon. With the improvement of preparation technology, some companies such as Element Six can produce high-quality synthetic diamonds with a few silicon impurities [13]. The identification characteristics of traditional CVD synthetic diamonds (basic gemological characteristics) are not enough to identify the CVD diamonds currently produced [14,15,16].

In addition, a systematic study of the basic gemological characteristics and spectroscopic characteristics of CVD synthetic diamonds is an effective way to identify and evaluate the quality of CVD synthetic diamonds. China can produce a large number of high-quality CVD diamonds, which play an important role in the international market. Accordingly, some colorless and near-colorless CVD diamonds produced by Zhengshi Technology Co., Ltd., China, were selected, and their gemological, spectroscopic, and luminescent characteristics were studied carefully. Additionally, color formation and influence factors of CVD synthetic diamonds were discussed compared to natural diamonds. Most natural diamonds are classified by Ia type for having aggregated nitrogen, while CVD synthetic diamonds are IIa without any detectable nitrogen. Thus, how to identify a natural diamond and CVD synthetic diamond is an important research topic in the field of gem identification.

## 2. Materials and Methods

### 2.1. Characteristics of Samples

**Figure 1:**
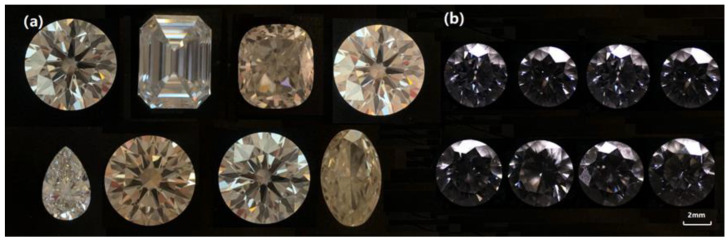
Appearance of diamond samples (**a**): CVD diamonds and (**b**): natural diamonds.

CVD synthetic diamond samples were produced by Zhengshi Technology Co., Ltd. (Shanghai, China). Gem-grade CVD synthetic diamonds were grown using the microwave plasma chemical vapor deposition (MPCVD) with the growth condition at temperature 1000 °C and pressure 14 kpa. Faceted CVD diamonds can be grown in size from 0.5 to 6.0 ct. Eight faceted CVD samples were used for further studies with colorless or near-colorless (see Table 1 and Figure 1a). The color grade ranges from E to K, the clarity ranges from VVS_1_ to SI_2_, and the weight of CVD samples ranges from 0.53 to 2.02 ct [12]. Meanwhile, eight faceted natural diamonds were selected (see Table 1 and Figure 1b) with color grading from D to K, the clarity ranging fromVS_2_ to SI_2_, and the weight ranging from 0.26 to 0.37 ct.

### 2.2. Methods

Color distribution was observed using a GI-MP22 binocular microscope (Baoguang Technologies, Nanjing, China). Strain patterns and anomalous birefringence were analyzed with a BX51 polarizing microscope (Olympus Corporation, Tokyo, Japan). Infrared spectra of all samples were tested byTensor-27 Fourier transform infrared spectrometer under room temperature, air humidity 11%, scanning range 4000–500 cm^−1^ in the mid-infrared band, equipment resolution 2 cm^−1^, average scanning time of background 30 times, and scanning average value of sample 60 times. Raman spectrometer (HORIBA, Kyuto, Japan) irradiated the sample with a specific wavelength of laser and recorded its Raman spectra. The solid-state semiconductor laser HORIBA, Kyuto, Japan) with 532 nm laser wavelengths and the type of HR-Evolution was used under the conditions at. Test range was 200–4000 cm^−1^, spectral resolution 3 cm^−1^, integration times 5, and scanning time 3 s.

The defects of samples were analyzed by UV-3600 spectrophotometer (Shimadzu, Kyuto, Japan). UV-vis spectra were acquired at room temperature, using high-speed scanning and transmission method (time constant 1.0 s, the average times 50, wavelength range 200–1000 nm, sampling interval 1.0). Photoluminescence (PL) spectra with 532 nm laser excitation using a Renishaw in Via Raman microscope (HORIBA, Kyuto, Japan) at room temperature were collected with some samples. Luminescent characteristics of samples under short wave fluorescence (265 nm) were observed under DiamondView (De Beers, London, UK). Optical, fluorescence, and phosphorescence images of samples were obtained by the testing software attached to the instrument. The adjustment of the field of view (FR) varies with the different shooting methods or the requirements for obtaining the internal characteristic information of samples. DiamondView can be used to observe color, intensity, and pattern of fluorescence and phosphorescence for analyzing the luminescent characteristics and growth structure of CVD synthetic diamonds and natural diamonds.

## 3. Results

### 3.1. Microscopic Examination

Both natural diamonds and CVD synthetic diamonds have the same refractive index, density, and appearance, and they have no distinct difference under a microscope. Obvious internal defects and stress lines might appear during the growth of natural diamonds due to the changes in external conditions [17,18,19].

**Figure 2:**
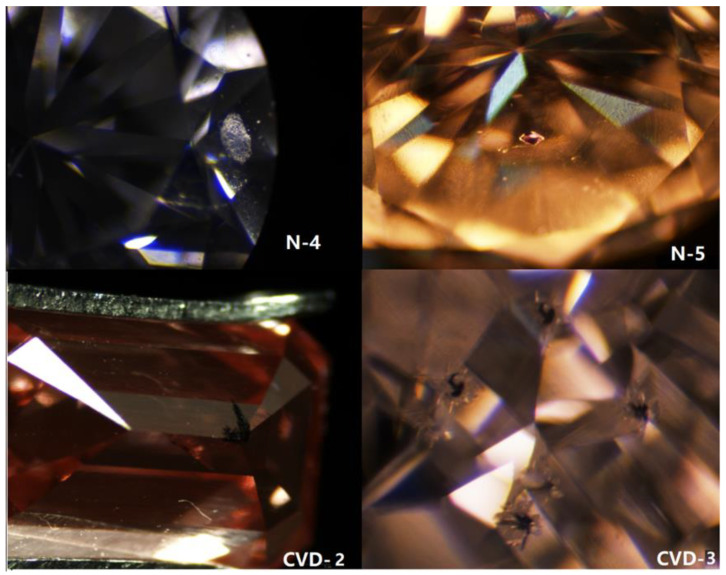
Different inclusions in CVD synthetic and natural diamond samples.

Point inclusions and a few feather cracks can be seen in natural diamond samples under a binocular microscope. Large white and red crystal inclusions can be seen in samples N-4 and N-5, respectively (Figure 2). By observing CVD synthetic diamond samples, it can be seen that some samples with low clarity contain point inclusions, feather inclusions, or black graphite inclusions. CVD-2 and CVD-3 samples both have obvious graphite inclusions (Figure 2), while natural diamond samples of N-4 and N-5 both have crystal inclusions (Figure 2). Sample N-4 has a white crystal (unidentified) inclusion under the table edge. Sample N-5 has a red crystal inclusion (pyrope, identified by Raman spectroscopy) directly under the table center. Thus, crystal inclusion is a typical identification of natural diamonds, and CVD synthetic diamonds always have graphite inclusions, especially in samples with low clarity grades.

**Figure 3:**
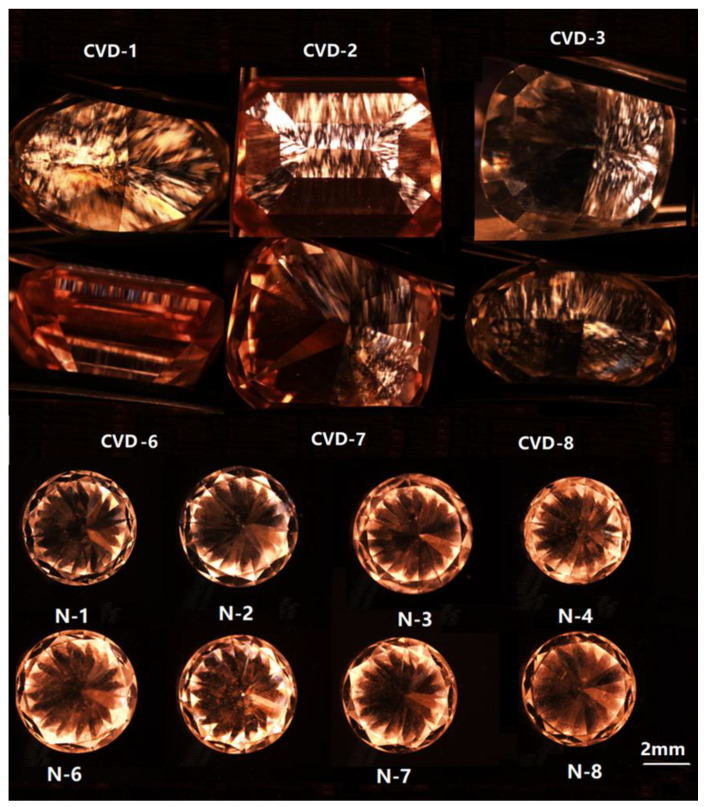
The interference and abnormal birefringence of samples.

Abnormal birefringence and random direction of interference color in natural diamonds can be observed under a polarizing microscope. However, CVD synthetic diamonds form stress lines with high-order interference color and obvious abnormal birefringence due to their short growth time [20]. This phenomenon can be observed with a polarizing microscope. All the CVD synthetic diamonds have grainy patchy birefringence patterns, which can be distinguished from natural diamonds. Compared to CVD samples in Figure 3, high color grade (E–F) of CVD-1, CVD-2, and CVD-3 has less abnormal birefringence and low interference color, while low color grade (I–K) of CVD-6, CVD-7, and CVD-8 has high abnormal birefringence and high order interference color. It can be inferred that internal defects and stress lines of diamonds influence their color grades. These grainy birefringence patterns, if clearly seen, are a very reliable criterion of recognition of CVD diamonds. Compared with CVD synthetic diamonds, natural diamonds have less abnormal birefringence and low interference color under the same magnification (Figure 3). CVD synthetic diamonds with the same color grade have more apparent abnormal birefringence than natural diamond samples and also have higher-order interference color than natural diamonds. This is helpful for distinguishing between CVD synthetic diamonds and natural diamonds.

### 3.2. Infrared Spectra

Nitrogen impurity in the diamond is inevitable due to the presence of nitrogen in the air. A small amount of nitrogen changes diamond’s color into yellow.

Nitrogen impurities will impact the color grade of CVD synthetic diamonds. In order to obtain diamonds with a high color grade, nitrogen will be controlled by manual intervention, such as synthesized under vacuum conditions, reducing growth rate, etc.

As to the technical problems, some factories need to use nitrogen to increase the growth speed during the synthesis process. Therefore, the characteristic absorption peaks related to nitrogen can be detected in CVD synthetic diamonds. However, many advanced synthetic methods can eliminate the impurity of nitrogen in CVD synthetic diamond [21], and the color grade is relatively high.

**Figure 4:**
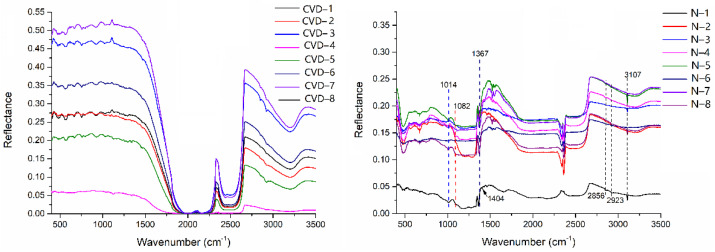
Infrared spectra of CVD synthetic diamonds and natural diamonds.

The infrared spectra of samples were detected by an infrared spectrometer, and the results were shown in Figure 4. The infrared absorption spectra of CVD synthetic diamonds show that there is no obvious characteristic absorption in the band of 1000–1400 cm^−1^. It can be concluded that all the samples are type IIa without detectable nitrogen. In the spectral range of 2700–3200 cm^−1^, several sharp lines of low intensity are found (Figure 4). They belong to hydrogen-related defects [8], so it can be inferred that CVD synthetic diamonds contain a few hydrogen impurities.

From Figure 4, it can be seen that besides sp^3^ hybrid orbital of carbon atom, natural diamond samples have the characteristic absorption peaks of 1014, 1082, and 1367 cm^−1^, which belong to aggregated nitrogen impurities. At the same time, 3107 cm^−1^ characteristic absorption peak related to hydrogen can be seen in all natural diamond samples, and 1404 cm^−1^ weak absorption peak can be seen in some samples (N-1, N-2, and N-3). When the 3107 cm^−1^ characteristic peak is weak, 1404 cm^−1^ characteristic peak does not appear, and these two characteristic absorption peaks are caused by C-H vibration absorption peaks; It can also be seen that the other two (2856 cm^−1^ and 2923 cm^−1^) characteristic absorption peaks related to the vibration absorption of H-C-H. The two absorption peaks of 2856 cm^−1^ and 2923 cm^−1^ always appear together, and 2923 cm^−1^ is always stronger than 2856 cm^−1^. As an important impurity element in diamonds, hydrogen has to go through a long geological evolution process in the process of forming hydrogen bond after entering the diamond lattice. Because the synthesis process takes a short time, hydrogen is not easy to enter the diamond lattice. Therefore, the existence of these absorption peaks can be used as one of the identification characteristics of natural diamonds [22]. However, CVD synthetic diamonds may have absorption peaks related to hydrogen produced by other companies [23]. Therefore, it can be concluded that all the natural diamond samples belong to the Ia type, while all the CVD synthetic diamond samples are IIa-type diamonds.

### 3.3. Raman Spectroscopy

**Figure 5:**
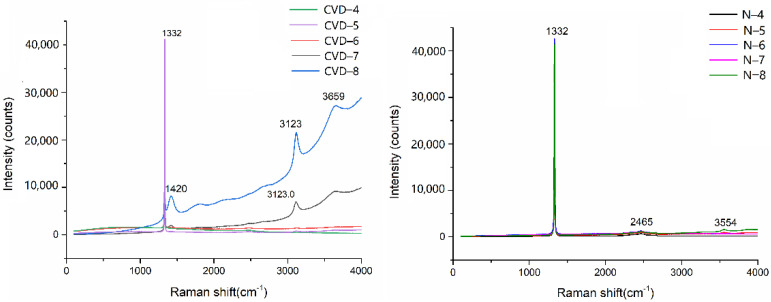
Raman spectra of low color grade (G–K) of samples.

Raman spectra of some samples with color grade (G–K) are shown in Figure 5. It can be seen that all the samples show a relatively single spectral line. Absorption spectra of faceted synthetic diamonds in the middle-infrared and near-infrared regions have no obvious absorption. An obvious carbon intrinsic peak at 1332 cm^−1^ is caused by the vibration of the sp^3^ hybrid orbital of carbon atom [24,25]. In addition to this characteristic peak, there is no other characteristic peak in faceted diamonds. Therefore, it can be speculated that there are almost no amorphous carbon or microcrystalline graphite inclusions in samples. Experimental results show that these samples are relatively pure and have fewer impurities. CVD-7 and CVD-8 samples have a slight peak of luminescence peak near 1420 cm^−1^, which is caused by the zero-phonon line of the 575 nm center (NV^0^) [26]. This is the reason why CVD-7 and CVD-8 samples have low color grades. Furthermore, there also have characteristic peaks of 3123 cm^−1^ and 3659 cm^−1^, which are zero-phonon line and the first vibrational replica of the NV^−^ center, respectively. They are luminescence usually exist in CVD synthetic diamonds related to nitrogen [27,28]. The intensity of luminescence is negatively correlated with the color grade of CVD diamonds.

Natural diamond samples have similar Raman spectra. Besides the first-order Raman scattering of 1332 cm^−1^, there are also weak characteristic peaks of 2465 cm^−1^ and 3554 cm^−1^. The feature at 2465 cm^−1^ is the second-order Raman scattering, and 3554 cm^−1^ is a kind of zero-phonon line luminescence, which needs to be further studied (Figure 5).

### 3.4. UV-Vis Spectra

**Figure 6:**
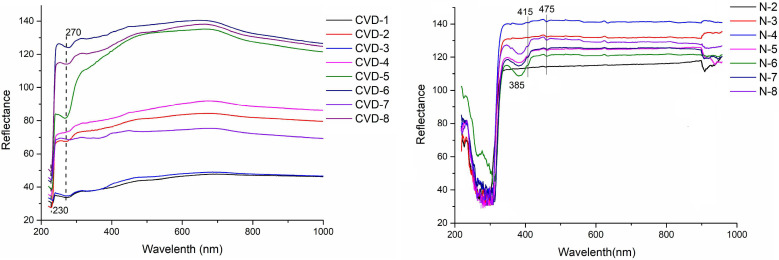
UV-Vis spectra of diamond samples.

UV-Vis spectra of all the samples were detected by a UV-Vis spectrophotometer. In addition to 230 nm, 270 nm absorption (related to isolated nitrogen) (see Figure 6), it can be observed there are no absorption peaks related to aggregated nitrogen such as 415 nm (N_3_ center), 452 nm, and 475 nm (N_2_ center) in all CVD synthetic samples. The UV-Vis spectra of CVD samples have shown little changes in the range of 300–700 nm, showing relatively flat spectra, and there is no strong absorption peak.

Unlike CVD samples, natural samples have a sharp absorption peak at about 300 nm. All the natural samples have weak absorption at 415 nm (N_3_ center) and 475 nm (N_2_ center) (Figure 6), indicating their natural origin. Notably, N-5, N-6, N-7, and N-8 samples have broad absorption lines at 385 nm, which is the electron-vibrational sideband of the N_3_ center [29]. Therefore, UV-Vis spectra of CVD samples differ from natural samples, and UV-Vis is a useful method to identify CVD diamonds.

### 3.5. PL Spectroscopy

PL spectroscopy is a sensitive analytical technique, which can detect lower concentrations of optical defects compared with UV-Vis absorption spectroscopy. Therefore, the PL method (where spectral features are excited by incident laser light) is capable of detecting optical defects in diamonds.

The PL spectra of low color grade samples are detected by a prompt photoluminescence signal centered at 532 nm, simultaneously with the UV pump pulse. The recorded photoluminescence signal shows some well-known characteristics, such as the charged silicon-vacancy center (Si-V) at 737 nm and the blue broadband luminescence centered at 448 nm [30].

Silicon is a characteristic impurity in CVD synthetic diamonds; it always appears in CVD growth and is rarely found in natural diamonds [31]. Silicon originates from the quartz windows on the CVD plasma chamber and enters the diamond lattice during its growth. The spectral characteristics related to silicon are strong evidence for the identification of CVD synthetic diamonds.

**Figure 7:**
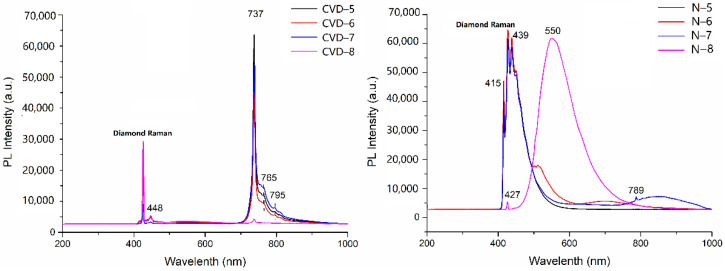
PL spectra of low color grade (H–K) diamond samples.

PL spectra of low color grade (H–K) diamond samples were tested, and the results were shown in Figure 7. CVD samples have 737 nm characteristic absorption peaks of silicon. Some CVD synthetic diamonds produced by Apollo and other factories have similar properties that the characteristic absorption peak of 737 nm is not easy to be detected [12]. Therefore, the existence of a 737 nm absorption peak can only be used as a reference for CVD synthetic diamonds, but it is not a conclusive characteristic to determine whether it is a CVD synthetic diamond. Besides the 737 nm absorption peak, there are two weak absorption peaks of 765 nm and 795 nm, probably also related to silicon [32,33,34].

Natural samples have the characteristic absorption peak at 415 nm (N_3_ center), and sample N-7 has a weak absorption peak at 789 nm. However, PL spectra of sample N-8 differs from other samples, which has a broad band of luminescence at 550 nm.

### 3.6. Fluorescence and Structural Characteristics under DiamondView

For the past two decades, the DiamondView fluorescence imaging instrument has been a core tool to determine the origin of diamonds [14]. The fluorescence reactions can be observed and examined in various orientations. Because of the very short wavelength and intensity of the ultraviolet excitation source, fluorescence is created in diamonds at locations just beneath facet surfaces, producing a distinct reaction. Although many CVD synthetics showed no reaction to a standard long-wave UV light source, all diamonds—including CVD synthetics—show some observable reaction to the high-intensity, high-energy UV source of the DiamondView.

In order to obtain the fluorescence color and intensity of diamond samples, DiamondView was selected to detect the luminescence of all samples. With the development of CVD synthetic diamond technology, the weight and quality of synthetic diamonds produced in different periods have increased significantly, and their luminescent characteristics are gradually changed. Early CVD synthetic diamonds without irradiation annealing usually show red or orange red fluorescence. In recent years, it has been found that the fluorescence color of the latest CVD synthetic diamond is close to that of natural diamond, and parts of CVD synthetic diamonds show a linear growth pattern under DiamondView [35].

**Figure 8:**
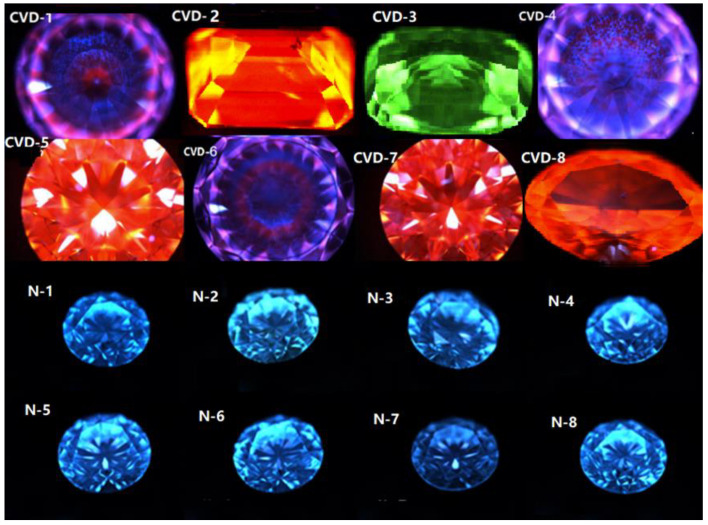
Fluorescence of CVD synthetics and natural diamonds under DiamondView.

Fluorescence color and intensity were observed under DiamondView. Most CVD samples produce orange red and blue, as well as the mottled distribution of purple, red, and blue (see Figure 8). At the same time, the phosphorescence of CVD samples is inert, while the samples of CVD-2, CVD-3, and CVD-4 show different degrees of weak blue-green phosphorescence. The different fluorescence color and phosphorescence intensity may be related to the growth batch of CVD synthetic diamonds. When the sample is rotated, the luminescent pattern is changed with different intensities, which is caused by the defects in the process of internal crystal plane sliding of CVD synthetic diamonds [36]. Some CVD diamonds show a distinct green appearance because of strong green fluorescence [37]. Sample CVD-3 has green fluorescence and shows a tiny green appearance.

Natural samples have blue fluorescence due to the N_3_ center, and the phosphorescence of all the samples is inert (Figure 8). Natural diamonds probably also have green fluorescence and H_3_ defect, similar to sample CVD-3. Thus, our observations indicate that this feature alone should not be used to determine a diamond’s natural origin.

**Figure 9:**
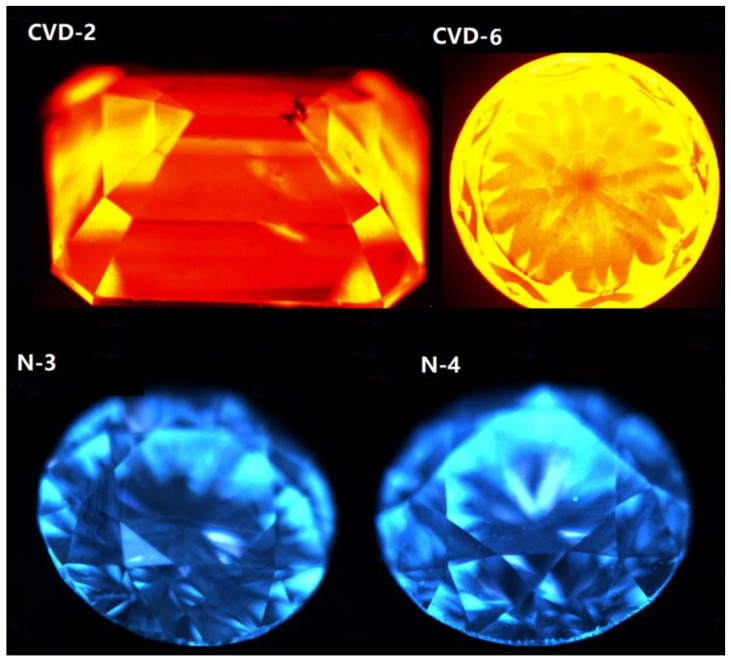
Different growth structures of samples under DiamondView.

The lamellar growth structure induced by some CVD samples can be observed (see Figure 9). According to the observation of the luminescent pattern, the sample has several different growth layers. The fluorescence of each layer is different, and most of them show orange red. The thickness of each layer is also slightly different, and the boundaries among layers are very clear.

Due to the existence of aggregated nitrogen in natural diamonds, the natural diamond samples show blue fluorescence. In addition, the growth structure of natural diamonds can be observed under DiamondView, e.g., irregular ring-like growth structure in N-3 (again see Figure 9). This special growth structure related to its growth condition, which is different from CVD diamonds.

## 4. Conclusions

Gemological characteristics of colorless and near-colorless CVD synthetic diamonds with different color grades produced by Zhengshi Technology Co., Ltd., Shanghai, China, were studied and compared with the same color grade of natural diamonds. All the CVD samples belong to type IIa and have little nitrogen, silicon, and hydrogen impurities, while natural diamonds are of type Ia. Colorless or near-colorless CVD diamonds have obvious abnormal birefringence and interference. A high color grade of CVD diamond has less abnormal birefringence and low-order interference, while a low color grade of CVD diamond has obvious abnormal birefringence and high-order interference color. UV-Vis spectra results show CVD diamonds have a small amount of isolated nitrogen, while natural diamonds have aggregated nitrogen and some low color grade samples have a small amount of hydrogen. PL spectra indicate that CVD diamonds have the characteristic absorption peaks of silicon, and natural diamonds have the absorption peaks of aggregated nitrogen. The colorless CVD diamonds show blue, orange red, purple red, and blue with mottled fluorescence, while natural diamonds show mainly blue fluorescence. CVD samples show lamellar growth and mottled luminescence pattern, and natural diamonds show irregular ring-like growth structure. So, multiple methods are needed to distinguish CVD synthetic diamonds from natural diamonds.

## Figures and Tables

**Table 1:** 4C classification of CVD diamond samples and natural diamond samples.

Label	Cut	Weight (ct)	Color	Clarity
CVD-1	Round	1.32	E	VVS_1_
CVD-2	Emerald	1.73	E	SI_2_
CVD-3	Pillow	1.00	F	SI_1_
CVD-4	Round	1.70	G	VVS_2_
CVD-5	Round	2.02	H	VS_1_
CVD-6	Round	2.01	I	SI_1_
CVD-7	Water drops	0.53	J	VS_2_
CVD-8	Olive	0.70	K	VVS_2_
N-1	Round	0.30	D	SI_2_
N-2	Round	0.33	E	VS_2_
N-3	Round	0.33	F	VS_2_
N-4	Round	0.26	G	SI_2_
N-5	Round	0.37	H	SI_2_
N-6	Round	0.34	I	SI_1_
N-7	Round	0.30	J	SI_1_
N-8	Round	0.32	K	SI_1_

## Data Availability

The data presented in this study are available on request from the corresponding author.
